# Development and internal validation of a nomogram for predicting short-term functional improvement after pharmacological treatment in severe symptomatic lumbar disk herniation

**DOI:** 10.3389/fmed.2026.1850770

**Published:** 2026-06-08

**Authors:** Hanze Mao, Guangqi Lu, Shuaiqi Zhou, Minghui Zhuang, Xin Xiu, Jing Li, Xinyue Sun, Yakun Liu, Mingming Ma, Jiaming Hu, Jie Yu, Liguo Zhu

**Affiliations:** 1 Wangjing Hospital, China Academy of Chinese Medical Sciences, Beijing, China; 2 Beijing Key Laboratory of Traditional Chinese Medicine Orthopaedic Techniques, Beijing, China

**Keywords:** functional improvement prediction, lumbar disk herniation (LDH), nomogram, Oswestry Disability Index (ODI), pharmacological treatment

## Abstract

**Background:**

Lumbar disk herniation (LDH) is an important cause of low back pain and functional impairment. For patients with severe LDH who are not immediate candidates for surgery or prefer to delay surgery, pharmacological treatment remains a major therapeutic option; however, short-term treatment responses vary substantially among individuals. At present, individualized tools for predicting short-term functional improvement after pharmacological treatment are lacking. Therefore, this study aimed to develop and internally validate a nomogram to predict 14-day improvement in the Oswestry Disability Index (ODI) in patients with severe LDH following pharmacological treatment.

**Methods:**

A total of 199 patients with MRI-confirmed severe LDH from 13 centers were included. All patients received 14 days of pharmacological treatment [Chinese patent medicine (CPM) vs. non-steroidal anti-inflammatory drugs (NSAIDs)]. The primary outcome was change in ODI at day 14. Candidate predictors were reduced using AIC-guided multivariable modeling, and a nomogram was developed from the final model. Secondary exploratory logistic analyses were performed using clinically relevant ODI improvement thresholds (>10, >20, and >30 points), with >10 points corresponding to the minimal clinically important difference (MCID). Internal validation was performed using bootstrap resampling.

**Results:**

The optimal linear prediction model included six key variables: treatment group, sex, alkaline phosphatase (ALP), angular instability, degree of disk herniation (DDH), and hypertrophy of the ligamentum flavum (HLF). In the primary linear model, CPM treatment, female sex, higher ALP levels, and DDH-protrusion were significantly associated with lower ODI improvement (all *P* < 0.05). In secondary threshold-based analyses, sex, ALP, and HLF were significant negative factors when ODI improvement was >30 points, while DDH-protrusion was associated with a lower likelihood of ODI improvement > 20 points. Calibration plots suggested acceptable agreement between predicted and observed 14-day ODI improvement in internal bootstrap validation.

**Conclusion:**

This internally validated nomogram may help estimate short-term functional improvement after pharmacological treatment in severe LDH and may assist pre-treatment risk stratification. However, given the short follow-up, limited sample size, and absence of external validation, the model should be considered preliminary and requires further validation before routine clinical use.

## 1 Introduction

With changes in modern lifestyle, the widespread presence of prolonged sitting, lack of exercise, and poor posture has led to a continuous increase in the incidence of low back pain (LBP) (1). According to statistics, there were approximately 619 million patients worldwide in 2020, and this number is expected to increase to 843 million by 2050 (2). Lumbar disk herniation (LDH) is one of the most common causes of LBP, affecting approximately 1%–3% of the population, with the majority of cases occurring between the ages of 30 and 50 (3). The main symptoms of the disease include lower back pain, radiating pain in the lower limbs, numbness, and muscle weakness. In severe cases, it can lead to the loss of normal functioning in daily life and work (4, 5). Especially in cases of severe LDH, patients often face intense pain and long-term functional impairment, making treatment options more complex (6).

For patients who have not yet met surgical indications, are temporarily unwilling to undergo surgery, or require short-term symptom control while awaiting further evaluation, pharmacological therapy remains an important component of conservative treatment for LDH (7–11). The commonly used drugs in clinical practice for LDH include non-steroidal anti-inflammatory drugs (NSAIDs), acetaminophen, muscle relaxants, and Chinese patent medicine (CPM) (8). NSAIDs are widely used for rapid symptom relief, but their use may be limited by gastrointestinal, renal, and cardiovascular adverse effects (9, 12). CPM is frequently used in routine practice in China and is supported by guideline-based and trial-based evidence as a potentially useful option in LDH management (13, 14). In particular, Yaobitong capsule has shown clinical efficacy and safety in patients with LDH and therefore represents a clinically relevant comparator in pharmacological treatment studies (15, 16). However, although these pharmacological options are commonly used in clinical practice, treatment response varies substantially between individuals.

Despite growing evidence on the average effects of different conservative and pharmacological strategies, existing studies mainly evaluate group-level treatment efficacy rather than individualized treatment response (17, 18). As a result, clinicians still lack practical tools to estimate which patients are more or less likely to achieve meaningful early functional improvement after a short pharmacological course. This gap is especially relevant in severe symptomatic LDH, in which short-term decisions may influence the need for intensified conservative care, closer monitoring, or earlier surgical reassessment.

Therefore, this study aimed to develop and internally validate a nomogram for predicting 14-day ODI improvement after pharmacological treatment in patients with severe symptomatic LDH using multicenter randomized trial data. We hypothesized that routinely available baseline demographic, laboratory, and imaging variables could be integrated into a clinically usable model for estimating short-term functional improvement.

## 2 Materials and methods

### 2.1 Study design

This study was a secondary analysis of patients with severe symptoms enrolled in a multicenter, parallel-group, superiority randomized controlled trial conducted in mainland China. From January 2023 to December 2024, patients were recruited from 13 participating centers, including Wangjing Hospital of China Academy of Chinese Medical Sciences, Dongzhimen Hospital of Beijing University of Chinese Medicine, and Guangdong Provincial Hospital of Traditional Chinese Medicine, based on the following inclusion and exclusion criteria. Written informed consent was obtained from all study participants.

### 2.2 Inclusion criteria

The participants were evaluated by spine specialists based on the guidelines of the North American Spine Society (NASS) “An evidence-based clinical guideline for the diagnosis and treatment of lumbar disk herniation with radiculopathy” (19):

1. Medical history: With or without a clear history of lumbar injury;
2. Symptoms and signs: LBP radiating to the buttocks and lower limbs, with pain worsening during increased abdominal pressure, such as coughing or sneezing; tenderness over the paraspinal area of the affected segment, radiating to the lower limbs, with restricted lumbar mobility; sensory hypersensitivity or dullness in the nerve distribution area of the affected lower limb, muscle atrophy in patients with a long course, and weakening of tendon reflexes, dorsiflexion strength of the great toe, etc.;
3. Physical examination: Motor or sensory deficits in the affected nerve; positive femoral nerve stretch test, positive straight leg raise (SLR) test, positive SLR with reinforcement test, or positive contralateral SLR test;
4. Auxiliary examinations: MRI indicating LDH, with the herniated segment corresponding to the clinical symptoms and signs.

The inclusion population consisted of patients aged 18–65 years who met the diagnostic criteria for LDH. Severe pain was defined as a leg pain visual analog scale (VAS) (20) score ≥7.

### 2.3 Exclusion criteria

1. History of spinal surgery;
2. Spinal compression fractures; lumbar spondylolisthesis grade II or higher; lumbar spondylolysis; lumbar spinal stenosis;
3. Spinal tumors; spinal tuberculosis; severe osteoporosis (T-score ≤−3.0 or associated fractures); diabetes with peripheral neuropathy;
4. Allergies to NSAIDs and CPM, or other related medications;
5. History of gastrointestinal ulcers/bleeding;
6. Patients undergoing coronary artery bypass surgery; those on dual-antiplatelet therapy or with coagulation disorders, or those at high risk of bleeding;
7. Severe skin diseases or skin lesions in the lumbar region;
8. Pregnant or breastfeeding women;
9. Severe heart failure, stroke, or other severe cardiovascular and cerebrovascular diseases; severe liver or kidney dysfunction;
10. Cauda equina injury, lower limb muscle strength ≤3, or persistent loss of lower limb motor or sensory function with indications for surgery;
11. Special populations unsuitable for clinical trials (e.g., blind, deaf, mute, or those with intellectual or mental disorders).

### 2.4 Selection of predictors

The demographic characteristics included gender, age, BMI, and occupation. The SLR test (21) was used as a physical examination sign for assessment. If one leg showed a positive reaction, it was considered positive; if both legs were negative, it was considered negative. The angle of the SLR was based on the positive side’s angle. If both sides were positive, the lower angle value was taken. The laboratory indicators included alanine aminotransferase (ALT), aspartate aminotransferase (AST), alkaline phosphatase (ALP), blood urea nitrogen (BUN), creatinine (Cr), and platelet count (PLT). The clinical assessment indicators included Oswestry Disability Index (ODI) (22)and 12-item Short Form Health Survey (SF-12) (23). The imaging parameters included: Scoliosis was diagnosed through X-ray anteroposterior images, with a Cobb angle greater than 10 degrees (24). Lateral images were used to assess lumbar lordosis, with the normal range being between 30 and 50 degrees (25). Angular instability was defined by a vertebral angle change greater than 10 degrees, while translational instability referred to vertebral displacement greater than 4 mm (26). MRI was used to assess ossification of ligamentum flavum (OLF), herniated disk with calcification (HDC), ossification of posterior longitudinal ligament (OPLL), spondylolysis, and spondylolisthesis. The degree of disk herniation (DDH) was classified based on the size and shape of the herniation, which included bulging, where the disk bulged within the intervertebral space; protrusion, where the disk extended beyond the intervertebral space but remained attached to the vertebral body; and extrusion, where the disk completely separated from the vertebral body (27). Sagittal herniation areas (SHA) and axial herniation areas (AHA) were used to quantify herniation. Hypertrophy of ligamentum flavum (HLF) was diagnosed when the ligament thickness exceeded 2 mm (28).

### 2.5 Interventions

Patients were randomly assigned to either the CPM group or the western medicine (WM) group. The CPM group received Yaobitong capsules plus placebo diclofenac sodium sustained-release tablets, whereas the WM group received diclofenac sodium sustained-release tablets plus placebo Yaobitong capsules. Both groups were instructed to rest in bed and wear lumbar support braces. Because each group received active treatment plus matched placebo, the treatment schedule followed a double-dummy design.

Treatment and outcome assessment were standardized over a 14-day period. Criteria for dropout, exclusion, and study termination included poor compliance, loss to follow-up, voluntary withdrawal, or serious adverse events.

### 2.6 Outcome definition

The primary outcome was absolute change in ODI score from baseline to day 14. Higher ODI improvement indicated greater short-term functional recovery.

Secondary exploratory analyses examined the probability of reaching three prespecified ODI improvement thresholds: >10, >20, and >30 points. The >10-point threshold corresponded to the Minimal Clinically Important Difference (MCID) used in the parent trial sample size calculation. Thresholds of >20 and >30 points were used to explore moderate and substantial short-term improvement, respectively.

### 2.7 Sample size

**FIGURE 1:**
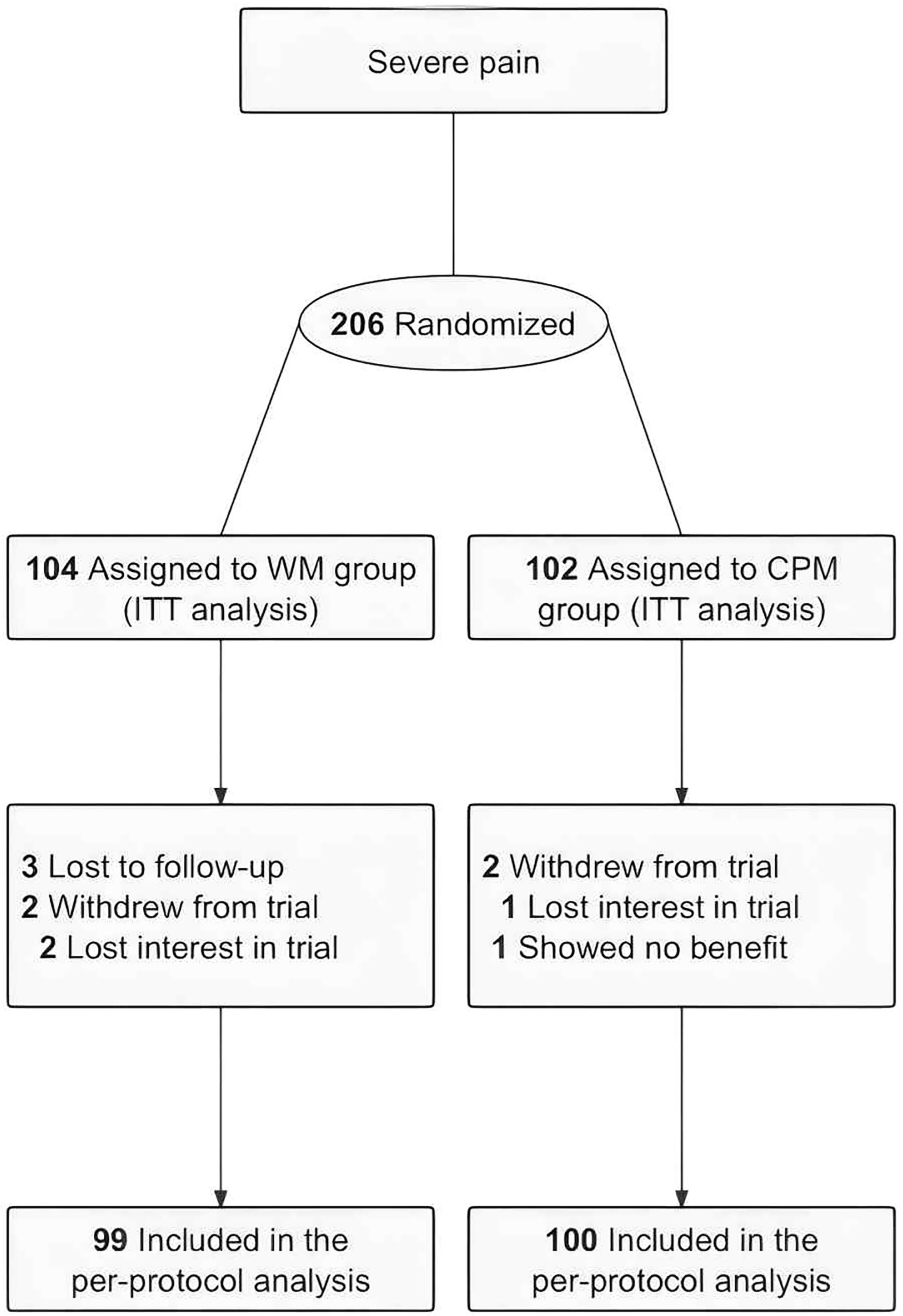
Participant flow diagram. This diagram shows the participant recruitment and progression throughout the study, detailing the inclusion and exclusion process, along with the total number of patients at each stage of the study.

*A priori* sample size calculations for the severe pain group were performed using R software (version 4.4.0). All calculations were based on detecting a Minimal Clinically Important Difference (MCID) of 10 points on ODI, with a standard deviation of 15.1 points derived from prior trial data (29). A statistical power of 90% and a two-sided alpha of 0.05 were assumed. The initial calculation indicated a requirement of 49 participants per arm. After accounting for a 20% dropout rate, the sample size was adjusted to 62 participants per arm. To ensure robust multi-center recruitment, the target sample size in the registered protocol was further increased to 108 participants per arm. In the final analysis, a total of 199 patients were included: 99 from the WM group and 100 from the CPM group (Figure 1).

### 2.8 Statistical analysis

Missing data were handled using multiple imputation by chained equations (MICE) (Supplementary Table 1). Continuous variables were imputed using predictive mean matching (PMM), while binary categorical variables were imputed using logistic regression. Five imputed datasets were generated, with 10 iterations each to ensure convergence. All analysis variables and relevant demographic covariates were included in the imputation model. Trace plots confirmed stable convergence across datasets (Supplementary Figure 1).

For the description of baseline characteristics, the Shapiro-Wilk Test was used to assess the normality of continuous variables. As the data were non-normally distributed, continuous variables with non-normal distribution were expressed as median and interquartile range (IQR), and intergroup comparisons were performed using the Kruskal-Wallis test. Categorical variables were presented as counts (percentages) *n* (%), and intergroup comparisons were conducted using the Chi-square test.

A full model strategy was applied for multivariable linear regression analysis to evaluate the independent associations of selected predictors with ODI improvement scores. Linear regression was chosen as the primary analysis because the outcome variable—ODI improvement score—is continuous, which allows direct estimation of the magnitude of functional improvement and more accurate assessment of predictor effects. Given the original study design was for treatment comparison and the sample size was relatively limited (*N* = 199), a bidirectional stepwise regression procedure based on the Akaike Information Criterion (AIC) was used to select variables, combining clinical relevance and statistical significance. Based on the events per variable (EPV) principle, each regression coefficient requires approximately 10–20 effective observations; the final model included six main effect variables, which exceeds the minimum recommended standard, suggesting that the sample size adequately supports model stability.

To further explore associations with different levels of clinical improvement, continuous ODI improvement scores were dichotomized based on three prespecified clinical thresholds (>10, >20, and >30 points) (12, 30). Multivariable logistic regression models were constructed for each threshold to analyze the independent association of each predictor with the probability of achieving the predefined improvement, with results reported as odds ratios (OR) and 95% confidence intervals (CI). Model discrimination was assessed using the concordance index (C-index), and optimal cutoff values were determined by maximizing the Youden index. Sensitivity, specificity, positive predictive value (PPV), and negative predictive value (NPV) were calculated for each threshold.

Based on the optimal linear prediction model, a nomogram was constructed. Internal validation was performed using the Bootstrap method, and a calibration curve was drawn to evaluate the prediction accuracy. All statistical analyses were performed using R software (version 4.5.1), with *P* < 0.05 considered statistically significant.

## 3 Results

### 3.1 Statistical description of baseline characteristics

The baseline characteristics (Table 1) was used to examine the associations between individual characteristics under two different treatment modalities. The Shapiro-Wilk test showed that all continuous variables did not meet the assumption of normality. Therefore, the chi-square test was used for categorical variables, and the rank-sum test was applied for continuous variables to assess the associations between baseline characteristics and treatment groups.

**TABLE 1:** Demographics and baseline characteristics of study participants.

Variable	Overall	WM group (*n* = 99)	CPM group (*n* = 100)	*P*
Age				0.648
Median (Q1, Q3)	47.0 (38.0, 58.0)	48.0 (38.0, 59.0)	47.0 (37.5, 57.0)	–
Gender				0.100
Male	89 (45%)	38 (38%)	51 (51%)	–
Female	110 (55%)	61 (62%)	49 (49%)	–
BMI				0.728
Median (Q1, Q3)	23.7 (21.8, 25.4)	23.5 (21.7, 25.0)	23.7 (22.0, 25.4)	–
Occupation				0.659
Both	38 (19%)	18 (18%)	20 (20%)	–
Mental	67 (34%)	36 (36%)	31 (31%)	–
Physical	43 (22%)	22 (22%)	21 (21%)	–
Retired	38 (19%)	19 (19%)	19 (19%)	–
Unemployed	13 (7%)	4 (4%)	9 (9%)	–
Income				0.900
<3000	54 (27%)	29 (29%)	25 (25%)	–
3,000–10,000	106 (53%)	52 (53%)	54 (54%)	–
10,000–20,000	35 (18%)	16 (16%)	19 (19%)	–
>20,000	4 (2%)	2 (2%)	2 (2%)	–
ALT				0.760
Median (Q1, Q3)	22.6 (14.6, 32.0)	23.0 (14.0, 32.0)	22.3 (15.0, 32.9)	–
AST				0.440
Median (Q1, Q3)	20.0 (16.3, 25.7)	20.9 (16.5, 26.0)	20.0 (16.0, 25.4)	–
ALP				0.695
Median (Q1, Q3)	72.0 (62.0, 86.0)	70.0 (62.0, 84.8)	72.0 (62.0, 89.2)	–
BUN				0.836
Median (Q1, Q3)	5.3 (4.3, 6.4)	5.5 (4.5, 6.3)	5.3 (4.3, 6.5)	–
Cr				0.060
Median (Q1, Q3)	66.1 (56.0, 75.0)	65.0 (52.0, 74.0)	68.2 (59.5, 76.5)	–
PLT				0.079
Median (Q1, Q3)	240.0 (206.0, 274.0)	250.0 (213.0, 281.0)	235.0 (200.0, 265.0)	–
VAS leg				0.787
Median (Q1, Q3)	7.0 (7.0, 8.0)	7.0 (7.0, 8.0)	7.0 (7.0, 8.0)	–
VAS LBP				0.675
Median (Q1, Q3)	7.2 (7.0, 8.0)	7.2 (7.0, 8.0)	7.5 (7.0, 8.0)	–
ODI (0)				0.947
Median (Q1, Q3)	60.0 (42.0, 70.0)	60.0 (44.0, 68.0)	57.0 (40.0, 70.0)	–
ODI (14)				0.174
Median (Q1, Q3)	32.0 (20.0, 46.0)	30.0 (20.0, 42.0)	34.0 (19.0, 52.0)	–
SLR angle				0.867
Median (Q1, Q3)	45.0 (35.0, 60.0)	45.0 (39.0, 60.0)	45.5 (35.0, 60.0)	–
SLR test				0.788
Impaired	181 (91%)	89 (90%)	92 (92%)	–
Normal	18 (9%)	10 (10%)	8 (8%)	–
PCS				0.307
Median (Q1, Q3)	28.8 (25.2, 35.1)	29.0 (25.5, 37.3)	28.4 (25.1, 34.1)	–
MCS				0.456
Median (Q1, Q3)	41.0 (35.9, 47.3)	41.4 (36.7, 47.0)	40.9 (34.6, 48.6)	–
Coronal scoliosis				0.244
Normal	186 (93%)	90 (91%)	96 (96%)	–
Impaired	13 (7%)	9 (9%)	4 (4%)	–
Lumbar lordosis				0.525
Normal	102 (51%)	48 (48%)	54 (54%)	–
Impaired	97 (49%)	51 (52%)	46 (46%)	–
Angular instability				0.988
No	190 (95%)	94 (95%)	96 (96%)	–
Yes	9 (5%)	5 (5%)	4 (4%)	–
Translational instability				>0.999
No	187 (94%)	93 (94%)	94 (94%)	–
Yes	12 (6%)	6 (6%)	6 (6%)	–
HDC				0.250
No	164 (82%)	78 (79%)	86 (86%)	–
Yes	35 (18%)	21 (21%)	14 (14%)	–
OPLL				0.985
No	186 (93%)	92 (93%)	94 (94%)	–
Yes	13 (7%)	7 (7%)	6 (6%)	–
Spondylolisthesis				>0.999
No	188 (94%)	94 (95%)	94 (94%)	–
Yes	11 (6%)	5 (5%)	6 (6%)	–
DDH				0.744
Bulging	27 (14%)	14 (14%)	13 (13%)	–
Protrusion	149 (75%)	72 (73%)	77 (77%)	–
Extrusion	23 (12%)	13 (13%)	10 (10%)	–
SHA				0.343
Median (Q1, Q3)	41.6 (21.7, 60.2)	44.7 (21.7, 64.2)	37.1 (21.3, 56.1)	–
AHA				0.754
Median (Q1, Q3)	47.7 (17.2, 78.2)	47.6 (17.1, 77.1)	47.9 (18.1, 79.4)	–
HLF				0.816
No	171 (86%)	84 (85%)	87 (87%)	–
Yes	28 (14%)	15 (15%)	13 (13%)	–
ODI improvement score				0.113
Median (Q1, Q3)	20.0 (12.0, 34.0)	22.0 (12.0, 36.0)	19.0 (10.0, 28.0)	–

There were no statistically significant differences between WM group (*n* = 99) and CPM group (*n* = 100) in demographic characteristics (sex, age, BMI, occupation, income), laboratory indicators (ALT, AST, ALP, etc.), imaging parameters (herniated disk with calcification, angular instability, degree of disk herniation, etc.), or clinical assessment indicators (SLR test, ODI, etc.) (all *P* > 0.05), indicating that the baseline characteristics of the two groups were overall balanced and comparable.

### 3.2 ODI scores and short-term functional improvement

At baseline, the median ODI score was 57.8 (95% CI: 53.8–61.8) in the CPM group and 57.5 (95% CI: 54.1–60.9) in the WM group, with no significant difference between groups (*P* = 0.73). At day 14 (Week 2), the median ODI score was 36.9 (95% CI: 32.8–41.1) in the CPM group and 32.2 (95% CI: 28.9–35.4) in the WM group. The between-group difference was 4.8 points (95% CI: 0.2–10.4, *P* = 0.04), indicating generally comparable short-term functional recovery, with the WM group showing slightly better improvement (Table 2).

**TABLE 2:** Oswestry Disability Index (ODI)^a^ scores at baseline and week 2 (intention-to-treat population).

Follow-up time	Mean (95% CI)			
	CPM (*n* = 100)	WM (*n* = 99)	Difference between groups	*P*-value
Baseline	57.8 (53.8 to 61.8)	57.5 (54.1 to 60.9)	0.3 (−5.9 to 4.2)	0.73
Week 2	36.9 (32.8 to 41.1)	32.2 (28.9 to 35.4)	4.8 (0.2 to 10.4)	0.04

*^a^*Oswestry Disability Index (ODI) assesses the effect of pain on normal daily activity including the ability to and intensity of lifting, care for oneself, walk, sit, sexual function, stand, social life, sleep and travel, ranging from 0 (no disability) to 100 (maximum disability possible).

### 3.3 Multivariable linear regression

In this study, a linear regression model combined with stepwise regression was used to construct a predictive model. The optimal model included treatment group, gender, ALP, angular instability, DDH, and HLF. The overall model was statistically significant (F = 3.721, *P* = 0.0008) with an adjusted R^2^ of 0.088, indicating that the selected variables explained approximately 8.8% of the variance in ODI improvement while controlling for model complexity. Multivariable linear regression analysis showed that treatment group (CPM Group vs. WM Group, β = −4.814, *P* = 0.028), gender (female vs. male, β = −4.614, *P* = 0.038), ALP (β = −0.092, *P* = 0.035), and DDH-Protrusion (β = −7.143, *P* = 0.020) were independent factors associated with ODI improvement scores. All these variables were significantly negatively correlated with ODI improvement, indicating that patients in CPM Group, female patients, those with higher ALP levels, and those with DDH-Protrusion tended to have relatively poorer functional improvement. HLF (β = −5.576, *P* = 0.078) and angular instability (β = −8.814, *P* = 0.098) also showed negative trends but did not reach statistical significance (Table 3). Sensitivity analyses of the linear model confirmed that the observed associations were robust and consistent with the main findings (Supplementary Table 2).

**TABLE 3:** Multivariable linear regression analysis of factors associated with Oswestry Disability Index (ODI) improvement.

Variable	*β* coefficient	Standard error	t-value	*P*
Group (CPM vs. WM)	−4.814	2.172	−2.217	0.028
Gender (female vs. male)	−4.614	2.209	−2.089	0.038
ALP	−0.092	0.043	−2.126	0.035
Angular instability	−8.814	5.305	−1.661	0.098
DDH-protrusion	−7.143	3.055	−2.338	0.02
DDH-extrusion	0.548	2.049	0.267	0.789
HLF	−5.576	3.144	−1.774	0.078

### 3.4 Exploratory threshold-based logistic regression analyses

The ODI improvement outcome was defined as a binary variable. The study set effective and ineffective standards based on different improvement thresholds (>10 points, >20 points, >30 points). For example, when the “improvement > 30 points” was set as the effective threshold, patients with an ODI score improvement greater than 30 points were assigned a value of 1 (effective), and those with an improvement ≤30 points were assigned a value of 0 (ineffective). Multivariable logistic regression models were established for each improvement threshold, systematically analyzing the association between various variables and the degree of ODI improvement at different levels.

The analysis results show that the impact of different factors on ODI improvement is heterogeneous. Gender, ALP, and HLF were found to be significant negative factors only when the highest improvement threshold (ODI > 30 points) was applied (OR = 0.410, 95%CI: 0.201–0.836, *P* = 0.014; OR = 0.982, 95%CI: 0.965–0.999, *P* = 0.033). DDH-Protrusion was already a significant negative factor at moderate or higher improvement levels (ODI > 20 points) (Table 4). Sensitivity analyses of the threshold-based logistic models confirmed that the observed associations were consistent with the primary analysis (Supplementary Table 3).

**TABLE 4:** Multivariable logistic regression analysis of Oswestry Disability Index (ODI) improvement at different thresholds.

Variable	ODI improvement (>100)		ODI improvement (>20)		ODI improvement (>30)	
	OR (95% CI)	*P*	OR (95% CI)	*P*	OR (95% CI)	*P*
Group (CPM vs. WM)	0.605 (0.307–1.192)	0.147	0.656 (0.356–1.211)	0.177	0.598 (0.297–1.205)	0.150
Gender (female vs. male)	0.604 (0.299–1.219)	0.160	0.721 (0.387–1.343)	0.303	0.410 (0.201–0.836)	0.014
ALP	0.991 (0.979–1.003)	0.145	0.975 (0.960–0.989)	<0.001	0.982 (0.965–0.999)	0.033
Angular instability	0.442 (0.107–1.826)	0.260	0.150 (0.016–1.370)	0.093	0.427 (0.047–3.889)	0.451
DDH- protrusion	0.941 (0.356–2.485)	0.902	0.347 (0.142–0.847)	0.020	0.270 (0.100–0.729)	0.010
DDH- extrusion	1.085 (0.570–2.067)	0.804	1.168 (0.651–2.095)	0.603	1.390 (0.718–2.688)	0.328
HLF	0.959 (0.368–2.501)	0.932	0.489 (0.205–1.164)	0.106	0.214 (0.048–0.960)	0.045

**FIGURE 2:**
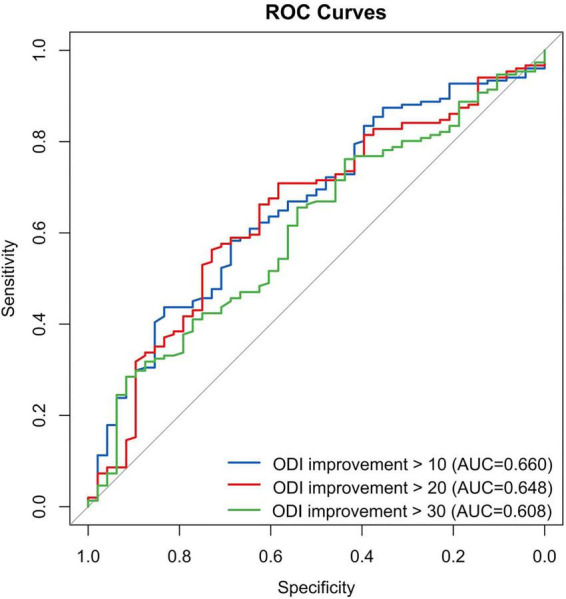
ROC curves for logistic regression models predicting different levels of Oswestry Disability Index (ODI) improvement. ROC curves depict the discriminatory performance of the logistic regression models for predicting ODI improvement at three thresholds: >10 points (blue line, AUC = 0.660), >20 points (red line, AUC = 0.648), and >30 points (green line, AUC = 0.608). The diagonal gray line represents the reference line for no discrimination (AUC = 0.5).

The analysis results show heterogeneous impacts of predictors, and overall model performance metrics at each ODI improvement threshold are presented in Figure 2. For an improvement of ≥10 points, the model had a C-index of 0.661 (95% CI: 0.575–0.746) with an optimal cutoff of 0.791 (Youden index), sensitivity 0.437, specificity 0.833, positive predictive value (PPV) 0.892, and negative predictive value (NPV) 0.320, indicating high specificity and PPV but limited NPV. For an improvement of ≥20 points, the C-index was 0.727 (95% CI: 0.657–0.797) with an optimal cutoff of 0.539, sensitivity 0.646, specificity 0.740, PPV 0.708, and NPV 0.673, suggesting good overall predictive performance for identifying patients with substantial functional improvement. For an improvement of ≥30 points, the C-index was 0.747 (95% CI: 0.671–0.823) with an optimal cutoff of 0.291, sensitivity 0.673, specificity 0.721, PPV 0.461, and NPV 0.862, indicating high reliability in ruling out patients who do not achieve marked improvement. Notably, at the ≥10-point threshold, most patients meet the criterion, resulting in a high PPV, whereas at the stricter ≥30-point threshold, fewer patients meet the criterion, leading to a higher NPV.

### 3.5 Nomogram

**FIGURE 3:**
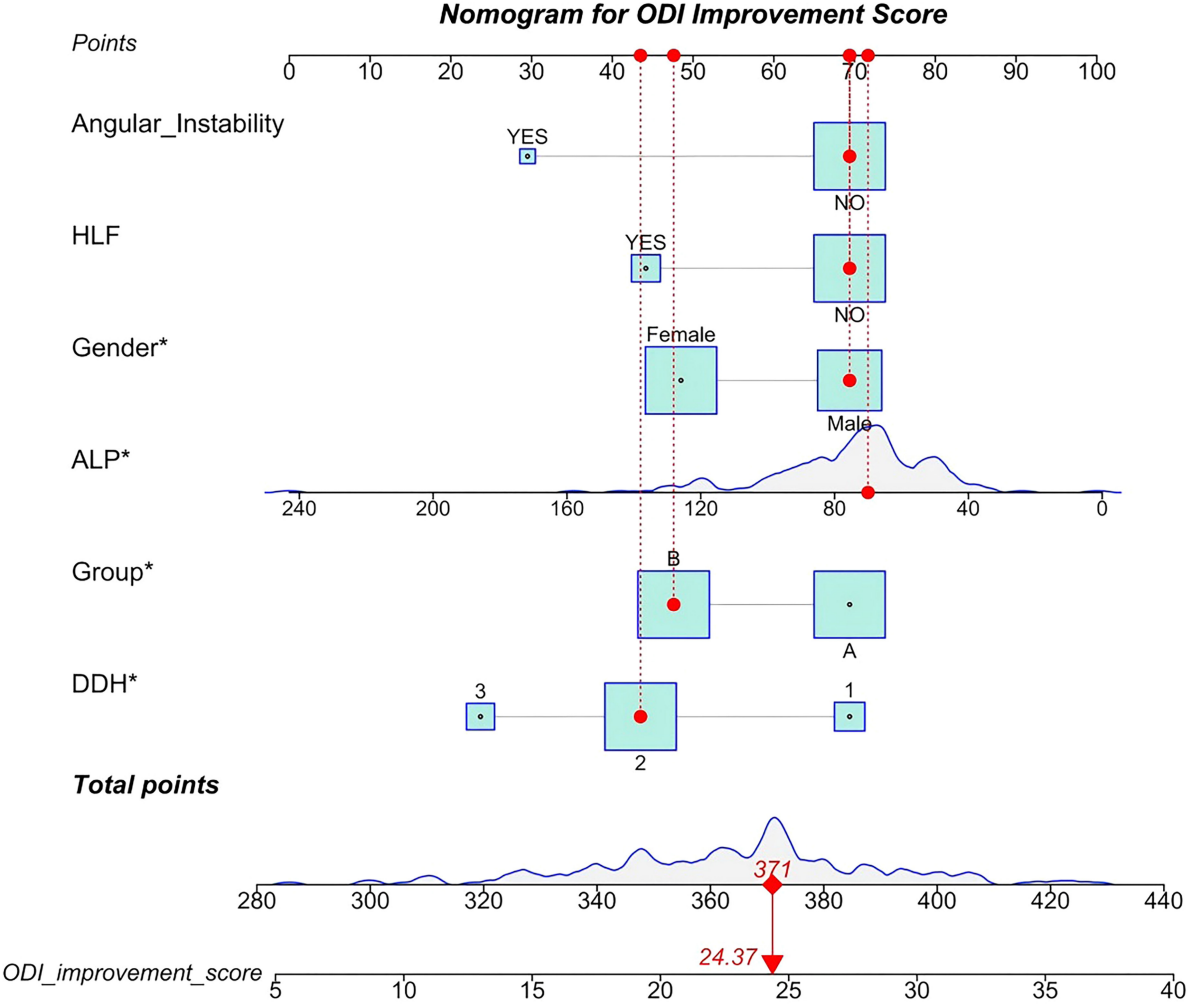
Nomogram for predicting 14-day Oswestry Disability Index (ODI) improvement score in patients with severe lumbar disk herniation (LDH). Points are assigned to each predictor based on its contribution to the model. Total points correspond to the predicted ODI improvement score. Predictors included: treatment group (WM vs. CPM), sex (male vs. female), alkaline phosphatase (ALP) level, angular instability (yes/no), degree of disk herniation (DDH) type (bulging, protrusion, extrusion), and HLF (yes/no). * Indicates predictors that were statistically significant in the multivariable regression model (*P* < 0.05).

The nomogram (Figure 3) integrates six key predictive variables and quantifies the probability of individual treatment effectiveness through a multivariable scoring system. The model enables personalized ODI score improvement assessment through an intuitive scale conversion. Each predictive variable is quantified using a score conversion system, with each variable assigned a point value between 0 and 100. The total score corresponds to ODI score improvement. For example, in a typical participant: a male with no angular instability, no HLF, treatment CPM group, with DDH-Protrusion, and an ALP of 70, the total score is 371 points, with an ODI score improvement of 24.37, which is very close to the actual improvement score (26 points), indicating good consistency between the assessment results and the actual improvement.

### 3.6 Calibration curve

**FIGURE 4:**
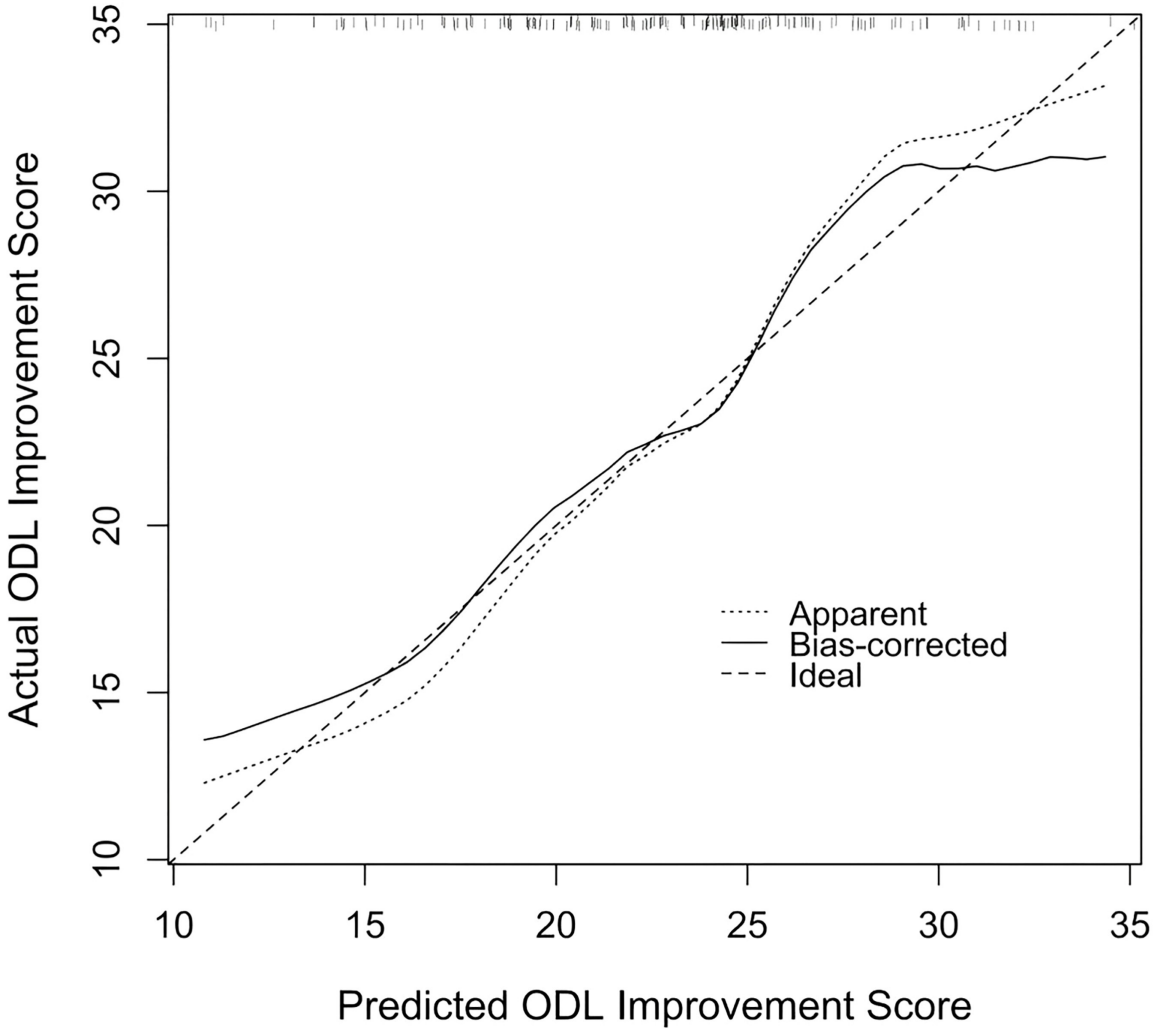
Calibration curve for the prediction model of 14-day Oswestry Disability Index (ODI) improvement. The x-axis represents the predicted ODI improvement score from the nomogram, and the y-axis represents the observed ODI improvement score. The solid line indicates perfect prediction (45-degree line), and the dashed line shows the model’s performance after internal bootstrap validation (1,000 resamples). The closer the dashed line is to the solid line, the better the model calibration.

The calibration curve (Figure 4) evaluates the accuracy of the model’s prediction of ODI improvement scores. The curve, after bootstrap correction, shows high consistency with the ideal curve across the entire prediction range, indicating that the model’s predicted values align well with the actual observed values, demonstrating excellent calibration.

## 4 Discussion

In this multicenter RCT-derived cohort, we developed and internally validated a nomogram to estimate 14-day ODI improvement after pharmacological treatment in patients with severe symptomatic LDH. The final model incorporated treatment group, sex, ALP, angular instability, DDH, and HLF. CPM treatment, female sex, higher ALP levels, and DDH-protrusion were associated with lower short-term functional improvement, while HLF and angular instability showed adverse trends within the final model.

The association between treatment group and ODI improvement may reflect differences in the short-term onset of pharmacological action. NSAIDs can provide relatively rapid analgesic and anti-inflammatory effects (31, 32), whereas CPM may exert a broader but potentially slower short-term effect profile (33). It should be noted that this observation should be interpreted cautiously, as the study was designed to develop a predictive model rather than to compare mechanistic superiority between drug classes. Specifically, the negative association of CPM with ODI improvement in this dataset reflects a predictive relationship and does not imply inferior efficacy.

The observed association between female sex and lower odds of substantial ODI improvement is consistent with prior literature suggesting sex-related heterogeneity in pain modulation, inflammatory response, and analgesic response (34, 35). Nevertheless, these explanations remain speculative in the context of the present dataset, and sex should be interpreted here as a predictor associated with short-term outcome rather than a causal determinant.

In this study, higher levels of alkaline phosphatase (ALP) were associated with lower short-term ODI improvement. ALP is an enzyme closely related to bone metabolism and bone formation, and its elevation may reflect more advanced degenerative or calcific changes in disk-related pathology, or abnormal bone metabolic activity. Previous studies have suggested that degenerative or calcified disk changes can compromise disk structural stability and cause persistent nerve root irritation, thereby delaying pain relief and functional recovery (36). However, this mechanism was not directly tested in the present study, and ALP should therefore be interpreted as a clinically available predictive marker rather than as a variable with causal significance.

Although the degree of disk herniation is generally positively correlated with the severity of symptoms, patients with DDH-Protrusion in this study showed poorer ODI improvement at moderate to high levels of improvement. Disk tissue at the herniation site can release inflammatory factors such as TNF-α and IL-1β over a long period, which triggers chemical irritation of the nerve roots and leads to persistent pain and functional impairment (37). Protrusion has limited contact between the nucleus pulposus and the immune system, resulting in insufficient macrophage infiltration (38). This leads to slow resolution of inflammation and prolonged absorption processes, which limits the effect of pharmacological interventions. Additionally, this study used ODI improvement scores as the outcome measure, and the extent of improvement is influenced by baseline functional impairment. For DDH-Extrusion extrusion patients, the higher baseline ODI scores provide a greater “potential for improvement,” allowing for more significant improvements.

Additionally, logistic regression showed that HLF has a significant negative impact on ODI improvement at the high improvement threshold, while angular instability only showed a trend. This suggests that imaging-based degenerative characteristics may have a potential impact on functional recovery: angular instability reflects poor segmental stability, which may lead to continuous mechanical irritation (39). HLF may exacerbate spinal canal stenosis, inhibiting the nerve root’s ability to recover (29). However, this interpretation also remain hypothesis-generating rather than causal.

From a practical perspective, the nomogram may be used at baseline to estimate the likelihood of short-term functional improvement after a 14-day pharmacological treatment course. Patients predicted to have limited improvement may benefit from closer follow-up, earlier multimodal conservative management, more careful counseling regarding expected short-term benefit, or earlier surgical reassessment if symptoms persist or worsen. In this sense, the model is best understood as a decision-support aid for early stratification rather than a stand-alone treatment decision rule.

Several negative or borderline findings also deserve emphasis. Not all retained predictors were statistically significant across all outcome thresholds, and the influence of predictors differed according to the level of ODI improvement examined. This pattern suggests that the determinants of achieving minimal clinically important improvement may differ from those associated with substantial short-term improvement.

## 5 Limitations

This study has several limitations. First, the analysis was based on a relatively small sample from a parent randomized trial designed for treatment comparison, not for prediction modeling, so overfitting and predictor instability cannot be excluded. Variable selection was guided by clinical rationale and the EPV principle, using stepwise regression to reduce model complexity; however, some optimism bias may remain, and the model should be considered hypothesis-generating, requiring validation in larger, multicenter cohorts. Second, only internal bootstrap validation was performed, and no external cohort was available, limiting generalizability. Third, follow-up was restricted to 14 days, so the model predicts only short-term functional improvement. Fourth, the AIC-based stepwise variable reduction was exploratory. Finally, several key prognostic factors, including symptom duration, neurological deficits, and psychosocial status, were not included, further limiting the model’s applicability. Future studies could also incorporate other non-surgical interventions such as physiotherapy or acupuncture to enhance generalizability.

## 6 Conclusion

We developed an internally validated nomogram to estimate 14-day ODI improvement after pharmacological treatment in patients with severe symptomatic LDH. The model integrates routinely available clinical, laboratory, and imaging variables and may assist early baseline risk stratification. However, given the short follow-up, limited sample size, exploratory modeling strategy, and lack of external validation, further studies are required before routine clinical implementation.

## Data Availability

The original contributions presented in this study are included in this article/Supplementary material, further inquiries can be directed to the corresponding authors.

## Supplementary material

Supplementary Figure 1 Trace plots for multiple imputation by chained equations (MICE). The plots show the mean and standard deviation of imputed variables across iterations, confirming stable convergence across the imputed datasets.
