# Supplementary material for: Development and internal validation of a nomogram for predicting short-term functional improvement after pharmacological treatment in severe symptomatic lumbar disk herniation
Source: Front Med (Lausanne). 2026 Jun 8;13:1850770. doi: 10.3389/fmed.2026.1850770 (PMC13283893; doi:10.3389/fmed.2026.1850770)
Supplement: Supplementary file 3 [file Table_3.docx]

| **Table S1. Overview of Missing Values for All Variables** | | | |
| --- | --- | --- | --- |
| **Variable** | **Total Sample** | **Missing (n)** | **Missing (%)** |
| LDH | 199 | 25 | 12.6 |
| SHA | 199 | 1 | 0.5 |
| AHA | 199 | 1 | 0.5 |
| HLF | 199 | 1 | 0.5 |

| **Table S2. Sensitivity Analysis of Multivariable Linear Regression Model** | | | | |
| --- | --- | --- | --- | --- |
| **Variable** | **β Coefficient** | **Standard Error** | **t value** | **P** |
| Group (B vs A) | -5.019 | 2.301 | -2.182 | 0.031 |
| Gender (Female vs Male) | -6.996 | 2.325 | -3.009 | 0.003 |
| ALP | -0.088 | 0.046 | -1.917 | 0.057 |
| BUN | -0.201 | 0.133 | -1.509 | 0.133 |
| PCS | -0.232 | 0.165 | -1.403 | 0.162 |
| Angular_Instability | -8.084 | 4.72 | -1.713 | 0.089 |
| DDH (2) | -11.206 | 3.444 | -3.254 | 0.001 |
| DDH (3) | 1.624 | 2.215 | 0.733 | 0.465 |
| HLF | -5.141 | 3.19 | -1.611 | 0.109 |

| **Table S3. Sensitivity Analysis of Threshold-Based Logistic Regression Models** | | | | | | |
| --- | --- | --- | --- | --- | --- | --- |
| **Variable** | **ODI Improvement （>10）** | | **ODI Improvement （>20）** | | **ODI Improvement （>30）** | |
|  | OR（95%CI） | P | OR（95%CI） | P | OR（95%CI） | P |
| Group (B vs A) | 0.655 (0.307–1.395) | 0.273 | 0.579 (0.286–1.174) | 0.129 | 0.530 (0.243–1.156) | 0.111 |
| Gender (Female vs Male) | 0.447 (0.202–0.990) | 0.047 | 0.431 (0.210–0.884) | 0.022 | 0.271 (0.120–0.611) | 0.002 |
| ALP | 0.992 (0.979–1.006) | 0.249 | 0.972 (0.955–0.990) | 0.002 | 0.981 (0.962–0.999) | 0.042 |
| BUN | 0.976 (0.939–1.014) | 0.207 | 0.957 (0.917–0.999) | 0.044 | 0.971 (0.930–1.013) | 0.180 |
| PCS | 0.977 (0.927–1.031) | 0.401 | 0.959 (0.911–1.009) | 0.108 | 0.979 (0.926–1.035) | 0.461 |
| Angular_Instability | 0.415 (0.110–1.564) | 0.194 | 0.247 (0.042–1.447) | 0.121 | 0.317 (0.034–2.975) | 0.315 |
| DDH (2) | 0.654 (0.192–2.232) | 0.499 | 0.117 (0.034–0.407) | 0.001 | 0.144 (0.044–0.472) | 0.001 |
| DDH (3) | 1.302 (0.606–2.796) | 0.499 | 1.435 (0.681–3.022) | 0.342 | 1.759 (0.843–3.673) | 0.132 |
| HLF | 1.240 (0.432–3.562) | 0.689 | 0.481 (0.187–1.239) | 0.13 | 0.188 (0.040–0.883) | 0.034 |
