# Supplementary figures and images for: Development and internal validation of a nomogram for predicting short-term functional improvement after pharmacological treatment in severe symptomatic lumbar disk herniation
Source: Front Med (Lausanne). 2026 Jun 8;13:1850770. doi: 10.3389/fmed.2026.1850770 (PMC13283893; doi:10.3389/fmed.2026.1850770)

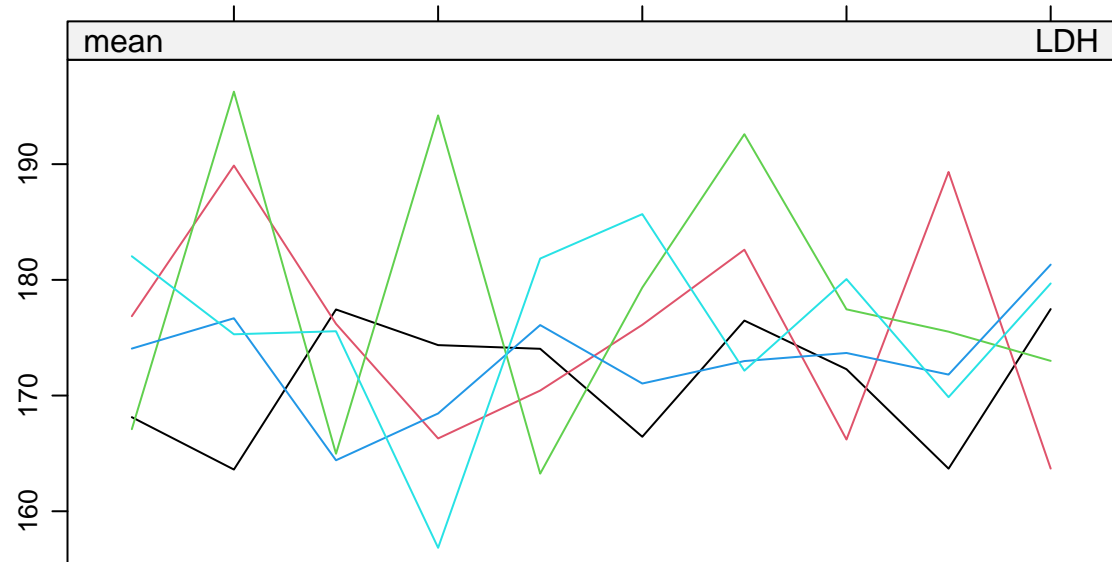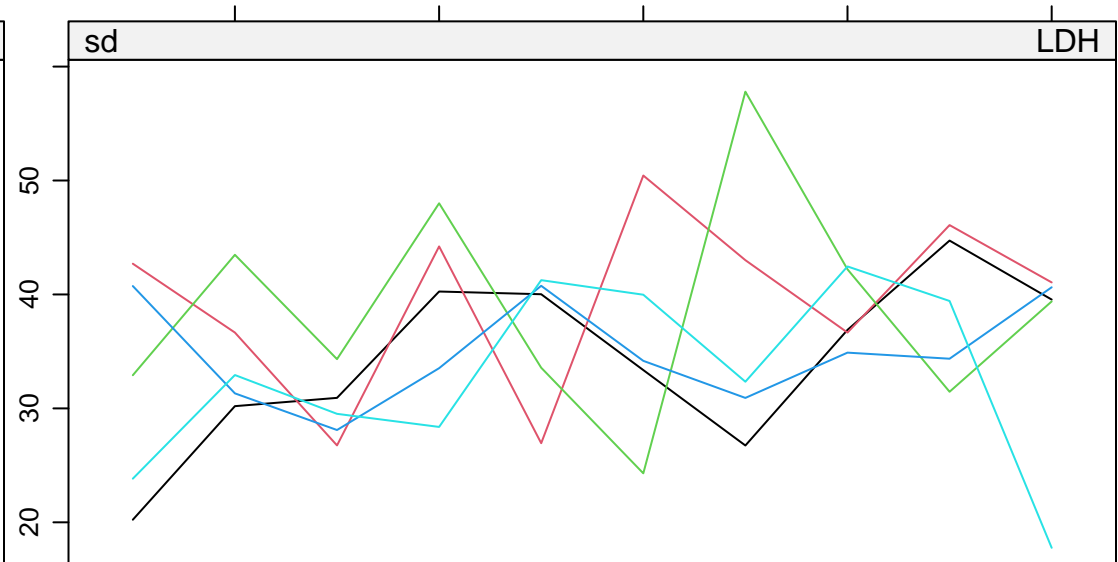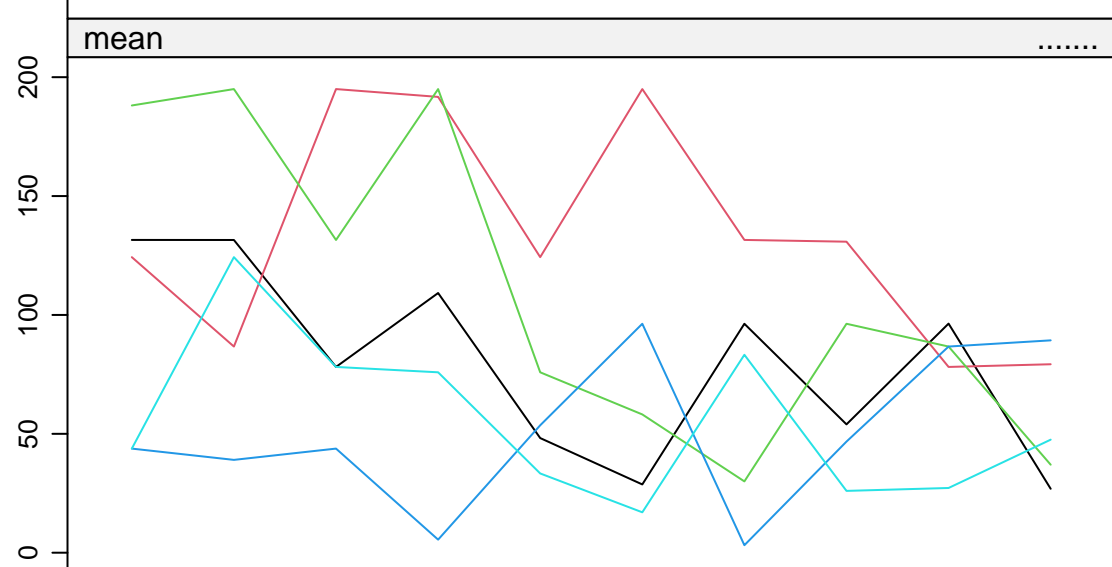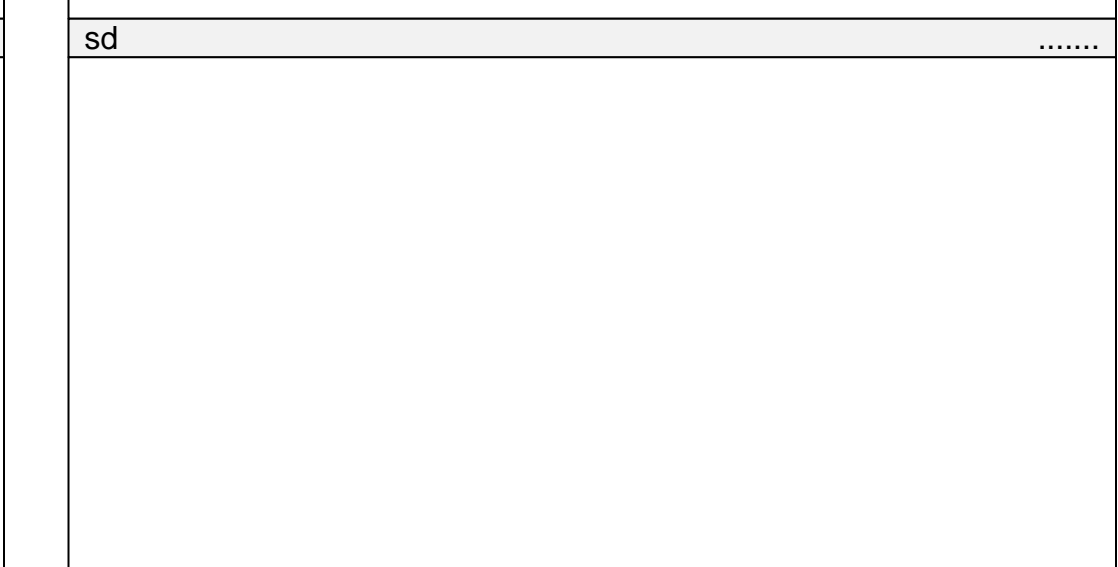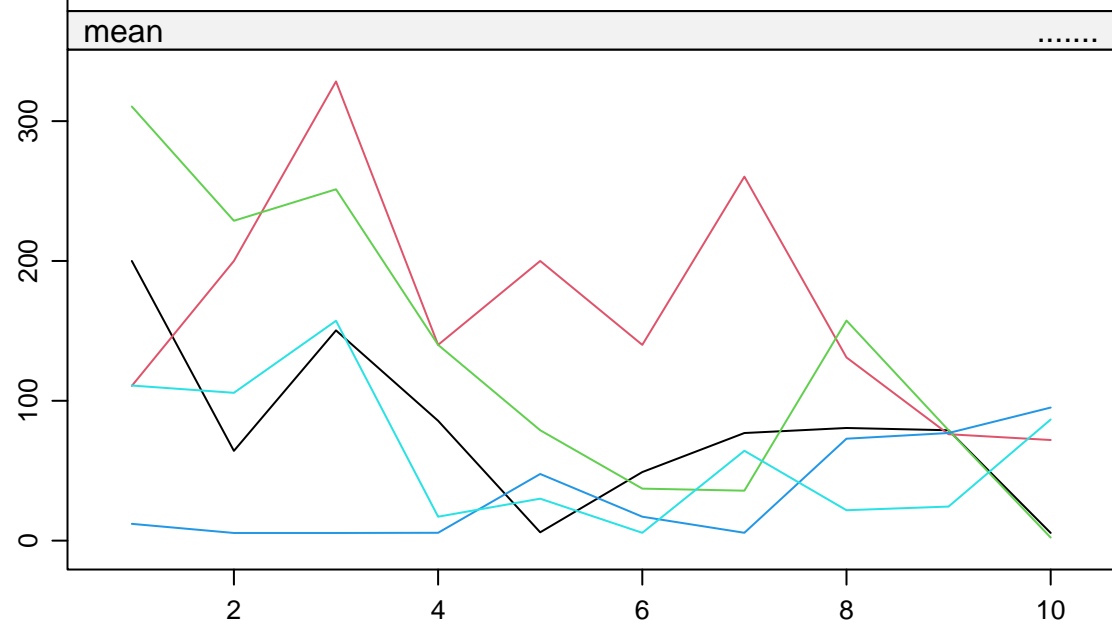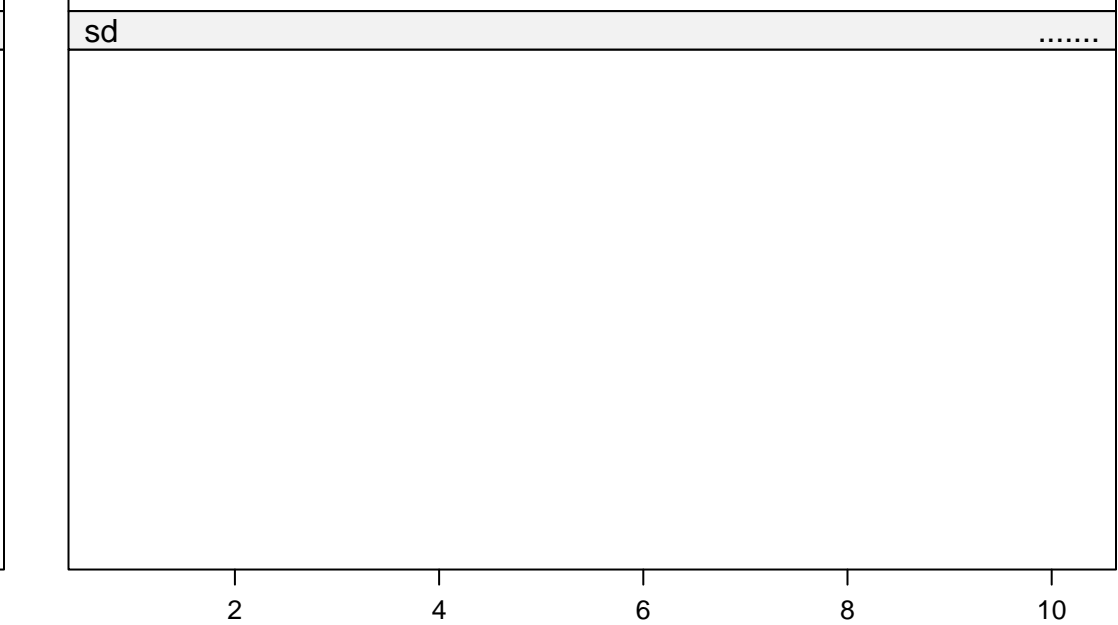

Iteration

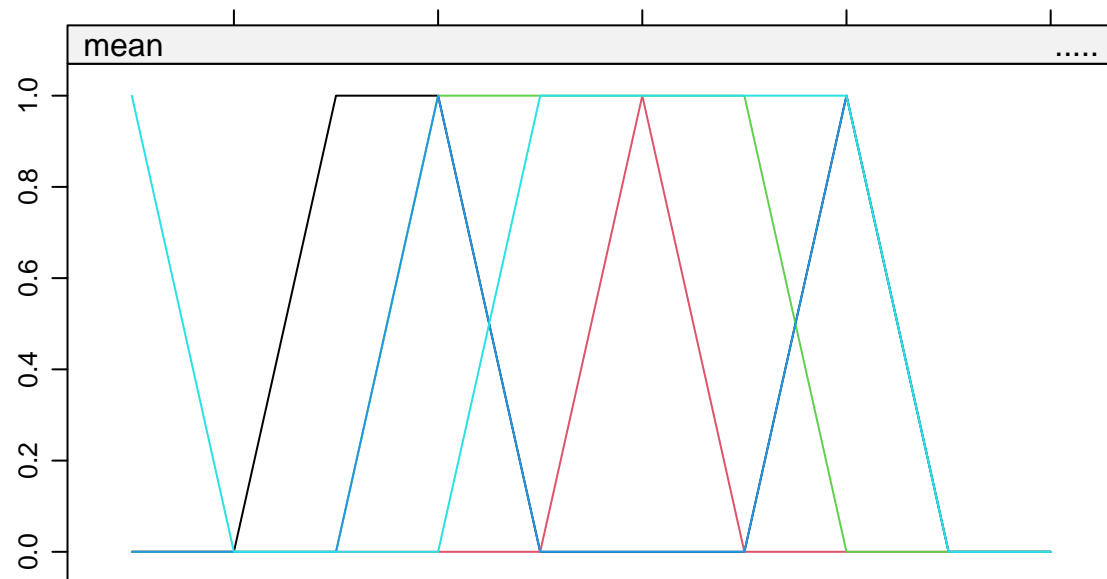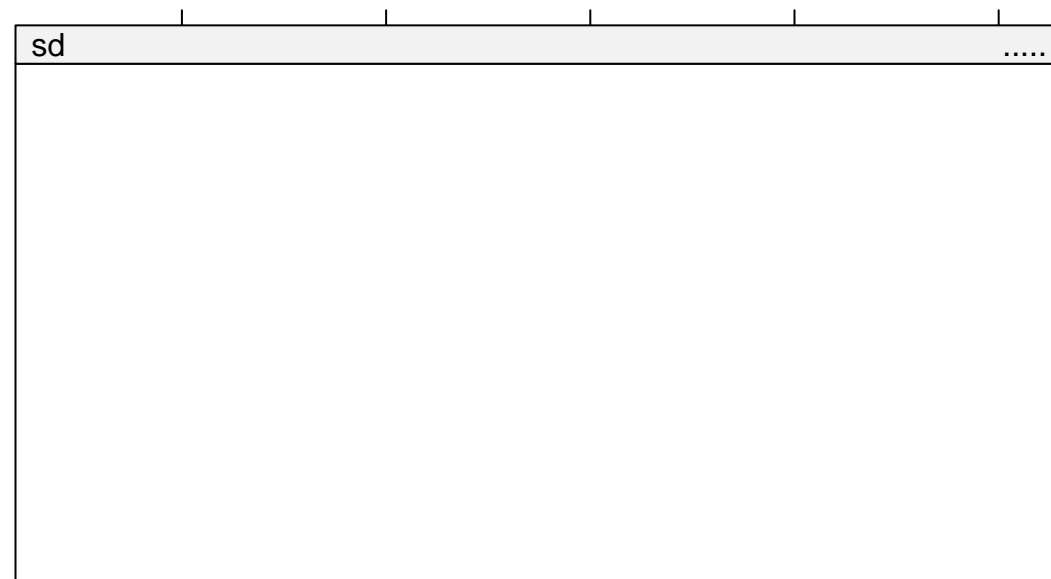

Supplement: Supplementary Figure 1 — Trace plots for multiple imputation by chained equations (MICE). The plots show the mean and standard deviation of imputed variables across iterations, confirming stable convergence across the imputed datasets. [file Data_Sheet_1.pdf]
